# Smoke on the Water: Comparative Assessment of Combined Thermal Shock Treatments for Control of Invasive Asian Clam, *Corbicula fluminea*

**DOI:** 10.1007/s00267-021-01474-x

**Published:** 2021-04-29

**Authors:** Neil E. Coughlan, Ross N. Cuthbert, Eoghan M. Cunningham, Stephen Potts, Diarmuid McSweeney, Gina Y. W. Vong, Emma Healey, Kate Crane, Joe M. Caffrey, Frances E. Lucy, Eithne Davis, Jaimie T. A. Dick

**Affiliations:** 1 Institute for Global Food Security, School of Biological Sciences, Queen’s University Belfast, 19 Chlorine Gardens, Belfast, BT9 5DL Northern Ireland UK; 2 Queen’s Marine Laboratory, Queen’s University Belfast, 12-13 The Strand, Portaferry, BT22 1PF Northern Ireland UK; 3 School of Biological, Earth and Environmental Sciences, University College Cork, Distillery Fields, North Mall, Cork, Ireland; 4 GEOMAR, Helmholtz-Zentrum für Ozeanforschung Kiel, Düsternbrooker Weg 20, 24105 Kiel, Germany; 5 INVAS Biosecurity Ltd., 82 Lakelands Close, Stillorgan, County Dublin, Ireland; 6 Centre for Environmental Research, Innovation & Sustainability, Institute of Technology Sligo, Ash Lane, County Sligo, Ireland

**Keywords:** Invasive alien species, Biosecurity, Open-flame heat torch, Thermal shock, Population control, Eradication

## Abstract

Suppression of established populations of invasive alien species can be a complex and expensive process, which is frequently unsuccessful. The Asian clam, *Corbicula fluminea* (Müller, 1774), is considered a high impact invader that can adversely alter freshwater ecosystems and decrease their socioeconomic value. To date, *C. fluminea* continues to spread and persist within freshwater environments worldwide, despite repeated management attempts to prevent dispersal and suppress established populations. As extensive *C. fluminea* beds can often become exposed during low-water conditions, the direct application of hot or cold thermal shock treatments has been proposed as suitable mechanism for their control. Further, mechanical substrate disturbance may enhance the efficacy of thermal shock treatments by facilitating exposures to multiple layers of buried clams. In the present study, we advanced these methods by assessing combined applications of both hot and cold thermal shock treatments for control of *C. fluminea*, using steam spray (≥100 °C; 350 kPa), low- or high-intensity open-flame burns (~1000 °C) and dry ice (−78 °C). In a direct comparison of raking combined with hot thermal shock applications, both steam and high-intensity open-flame treatments tended to be most effective, especially following multiple applications. In addition, when hot thermal treatments are followed by a final cold shock (i.e. dry ice), steam treatments tended to be most effective. Further, when dry ice was applied either alone or prior to an application of a hot shock treatment, substantial if not complete *C. fluminea* mortality was observed. Overall, this study demonstrated that combined applications of hot and cold thermal shock treatments, applied following the disruption of the substrate, can substantially increase *C. fluminea* mortality compared to separate hot or cold treatments.

## Introduction

Invasive alien species are considered a key component of global change, as established invader populations can detrimentally alter ecological and evolutionary dynamics, which in turn can negatively impact ecosystems and decrease their socioeconomic value (Sousa et al. 2009; 2014; Crane et al. 2020). In general, management options for suppression and eradication can be complex undertakings, which are often costly and resource intensive to preform (Caffrey et al. 2011b; Wittmann et al. 2012a, b; Piria et al. 2017). Further, the efficacy of many control techniques are unknown, inadequate and damaging to non-target species (Wittmann et al. 2012a, b; Caffrey et al. 2014; Sousa et al. 2014). Accordingly, there is a clear and urgent need to develop appropriate and readily available control methods, which maximise efficacy of treatment towards target species, but minimise broad-scale environmental damage (Coughlan et al. 2018a, b; Cuthbert et al. 2018; 2019). Ideally, any such method should be simple and relatively straightforward to apply within field scenarios (Coughlan et al. 2018b). This is especially important as the implementation of control strategies can be impeded by numerous obstacles, including lack of public awareness and engagement (Davis et al. 2018), poor enforcement of regulations, inadequate monitoring and rapid-response protocols (Caffrey et al. 2014) and legal barriers, such as accessibility of private property (Piria et al. 2017). In addition, more traditional methods of invader control, such as chemical treatments, are no longer available due to legislative changes in light of undesirable environmental or human-health effects or are simply inappropriate or ineffective for large, open waterbodies rather than industrial settings (Sousa et al. 2014).

The Asian clam, *Corbicula fluminea* (Müller, 1774), is a high impact invasive bivalve, which is considered a major threat to the function and biodiversity of freshwater ecosystems worldwide (Sousa et al. 2008; 2014). As a dominant filter feeder, *C. fluminea* can homogenise macroinvertebrate communities, physically alter benthic habitats and disrupt ecosystem-regulating services (McMahon 1982; Sousa et al. 2008; 2014). Equally, its presence can have substantial negative economic impacts, through macrofouling of agricultural, municipal and raw water extraction systems, increased sedimentation rates and the closure of sport fisheries and amenity areas (Nakano and Strayer 2014; Sousa et al. 2014). Moreover, *C. fluminea* has shown a high degree of physiological and ecological plasticity (Sousa et al. 2014), and an exceptional capacity for human-mediated or even zoochorous dispersal (Belz et al. 2012; Coughlan et al. 2017b). Despite repeated management efforts to curtail invader spread, *C. fluminea* continues to spread across hydrologically unconnected freshwater systems (Barbour et al. 2013; Caffrey et al. 2016; Colwell et al. 2017). Further, at the current predicted rate of climate change, novel river basins will be increasingly at risk of invasion as new areas of suitable habitat become available, especially at higher latitudes (Gama et al. 2017).

Once established, *C. fluminea* can form dense and expansive populations that are notoriously difficult to eradicate or control (Caffrey et al. 2011a; Wittmann et al. 2012a, b). For example, although extensive eradication and control experiments can achieve a short term reduction of both *C. fluminea* density and biomass, none have successfully provided a substantial long-term management solution for *C. fluminea* populations (Wittmann et al. 2012a, b; Sheehan et al. 2014). Accordingly, there is an urgent need to develop, refine and validate tools capable of providing rapid yet long-term control and eradication of emerging and existing *C. fluminea* populations (Colwell et al. 2017; Coughlan et al. 2018b; 2019b; 2020).

Recently, through a series of laboratory experiments, Coughlan et al. (2018b) observed that cold thermal shock treatments, caused by an application of dry ice pellets (i.e. solid CO_2_ pellets at −78 °C), could be used to kill tidally exposed, substrate-residing *C. fluminea*. Similarly, Coughlan et al. (2019b) demonstrated that open-flame torch (~1000 °C, i.e. hot thermal treatments) can also be used to kill mud-dwelling *C. fluminea*. Building on this, Coughlan et al. (2020) showed that a continuous jet of steam (≥100 °C) can likewise be used as an effective tool for substantial population control of low-water exposed bivalves. Overall, when taken together, these studies show the usefulness of individual thermal shock treatments as methods for effective, rapid-response control and possible eradication of *C. fluminea* populations (Coughlan et al. 2020). In particular, thermal shock treatments potentially represent a straightforward, user and environmentally friendly mechanism for causing substantial if not complete mortality of invasive alien bivalve species residing upon and within exposed lake, river or canal beds (Crane et al. 2019; Joyce et al. 2019; Coughlan et al. 2020). Notably, Coughlan et al. (2019b) observed an increased efficacy for thermal shock treatments when applied following the mechanical disruption of the structural integrity of the substrate, whereby the substrate was raked and churned to expose buried *C. fluminea*. Following the work of Coughlan et al. (2019b; 2020), we suggest that combined approaches require investigation, whereby multiple applications of various control mechanisms could be combined and strategically applied to increase overall bivalve mortality. Further, dry ice applications may increase mortality if the penetration of a cold thermal shock is greater than hot thermal applications, through freezing of the substrate surrounding *C. fluminea*. In particular, a sudden change of temperature that prevents any chance of acclimation, especially over large temperature gradients, will likely increase mortality rates due to escalated thermal shock (e.g. McMahon and Ussery 1995). As such, combined applications of dry ice and hot thermal treatments may increase overall mortality, with potential to reduce time and labour costs by decreasing the frequency and duration of hot thermal exposure periods. Accordingly, the application of a cold or hot thermal shock treatment immediately following the application of a contrasting hot or cold treatment will likely be more destructive. Further, such alternation of hot and cold thermal treatments could reduce the intensity and/or duration of treatment needed to kill substrate dwelling specimens of *C. fluminea*.

First, using simulated bivalve beds, we ascertained the effectiveness of both reduced intensity and exposure time without the addition of a cold thermal shock (i.e. dry ice). To achieve this, we comparatively assessed the combined effects of substrate disruption, i.e. raking, paired with various rapidly applied hot thermal shock treatments, which consisted of steam, low- or high-intensity open-flame burns without the addition of a cold thermal shock. The effects of raking combined with hot thermal shock treatments, followed by a final application of cold thermal shock that was delivered via the application of dry ice, was also examined. Similarly, the inverse of the process was likewise assessed, whereby following substrate raking, a cold thermal shock treatment was immediately applied, followed by the application of a hot thermal shock. A temperature gradient of cold to hot is particularly interesting as many bivalve species show greater tolerance for sudden decline rather than a rapid increase in temperatures (e.g. presence of haemolymph ice-nucleating proteins; Madison et al. 1991). Therefore, a sudden upwards elevation of temperature is expected to be more damaging than accelerated cooling, particularly at a sub-lethal cellular level (e.g. Hicks and McMahon 2002). Overall, we hypothesised that exposure to the more extreme heat delivery of an intense open-flame burn would result in greater *C. fluminea* mortality than steam or low-intensity burns. Similarly, it was expected that the effect of combined hot and cold thermal shock treatments would increase overall bivalve mortality rates. Thus, we hypothesised that cold followed by hot thermal shock applications would cause even greater *C. fluminea* mortality than application of hot followed by cold shock treatments, given that a warmed substrate could inhibit its penetration by extreme cold. Thus, through a series of factorial experiments, we assessed potential avenues for the further development of thermal shock treatments as a means of rapid-response invader control.

## Methods

### Specimen Collection and Maintenance

Specimens of *C. fluminea* were collected from the extensive, tidally exposed area at Poulmounty on the River Barrow in the Republic of Ireland (52°29′15.11″N, 6°55′42.20″W) during May 2019, and transported in source water to Queen’s University Marine Laboratory, Northern Ireland. In the laboratory, specimens were kept within a controlled temperature (CT) room at 13 °C, on a 12:12 h light-to-dark schedule. All specimens were maintained in aerated aquaria using locally sourced lake water (Lough Cowey: 54°24′41.79″N, 5°32′25.96″W). Specimens were allowed to acclimatise to these conditions for at least 1 week prior to experimentation. Further, only living and feeding specimens were selected for experimental work, i.e. selected specimens that were observed opening to feed, and reclosed when disturbed. Adult *C. fluminea* specimens were selected by shell height (SH), i.e. from the highest point on the umbo to the ventral margin of the shell ‘umbo to gape’.

### Experiment 1: Effect of Raking with Hot Thermal Shock Treatments

To mimic field scenarios, whereby *C. fluminea* are found residing in low-water exposed substrate, specimens (SH min.–max.: 18–20 mm) were encapsulated within damp sand patches. Groups of 30 *C. fluminea* were randomly mixed into a damp sand layer to create each simulated patch, which is representative of a realistic *C. fluminea* bed structure (25 cm × 25 cm; *~*4 cm deep: 480 ind. m^−2^). Combined applications of rake and thermal shock treatments were then examined, further building on the results of Coughlan et al. (2019b; 2020), who identified that joint rake and thermal shock treatments can kill substrate-residing *C. fluminea*. In particular, much of the experimental set-up deployed by Coughlan et al. (2020) was also used by the present study to enable comparison between these works. The initial raking phase was used to churn-up and furrow the substrate, to expose greater numbers of *C. fluminea* to the subsequent thermal shock treatments. Specimens were exposed to a continuous jet of steam (≥100 °C; 350 kPa; Karcher^®^ SC3 Steam Cleaner), a low-intensity open-flame burn (~1000 °C; ASAB weed-burner AS-09463: butane gas) or high-intensity open-flame burn (~1000 °C, 400 kPa: Rothenberger, Romaxi Power Burner: butane gas) for a 2.5-min period, following a 30-s period of patch raking (Fiskars soil rake). A thermal exposure duration lasting 2.5 min was selected as 2- and 3-min exposure periods have previously been observed to deliver high but not complete mortality of *C. fluminea* (Coughlan et al. 2020). The low-intensity torch had a shorter ‘blue-flame’ length than the high-intensity device, i.e. a shorter hottest burning section of flame, at ~5 and ~36 cm, respectively. The combined applications of rake and thermal shock were examined for single, double or triple treatments (*n* = 3 per experimental group). Treatments conducted in the absence of raking were not considered for Experiment 1, as results from this approach have already been adequately demonstrated in Coughlan et al. (2019b; 2020). Control groups were likewise formed into sand patches, which were each raked for up to three consecutive 30-s periods and allowed to air dry for a 2.5-min period following each raking event. Control patches were not exposed to thermal shock treatments. Following a 15-min cooling period, initiated after the final thermal shock treatment had occurred, specimens were immediately extracted from the patch and returned to the CT room. Replicates were then placed individually within 600 ml of dechlorinated tap water taken from a continuously aerated source (11–13 °C) for a 24-h recovery period, after which mortality was assessed. Specimens were considered dead if they were gaping, or failed to respond to a tactile stimulus, or did not reclose (see Matthews and McMahon 1999).

### Experiment 2: Effect of Raking with Hot Thermal Shock Treatments, then a Final Cold Shock Application

To assess the combined efficacy of combined hot and cold thermal shock treatments to kill sand dwelling *C. fluminea*, specimens were factorially exposed to treatments under both non-disrupted substrate and disrupted substrate (i.e. non-raked or raked). This was immediately followed by the application of a hot thermal shock, followed by a single final cold thermal shock. Hot thermal shock treatments consisted of steam spray, a low-intensity open-flame burn or high-intensity open-flame burn (see above). Cold thermal shock was delivered using 9 mm dry ice pellets, (i.e. solid CO_2_ pellets at −78 °C). As described above, groups of 30 *C. fluminea* specimens (SH min.–max.: 19–21 mm) were mixed into damp sand patches. Following an initial 30-s non-raking or raking period, specimens were exposed to steam or burn treatments for a 2.5-min period. For rake-treated groups, all combinations of experimental applications were examined for single, double or triple treatments (*n* = 3 per experimental group). Immediately following the application of the final hot thermal shock treatment, 800 g of dry ice was evenly applied to the upward facing surface area of the patch, for a 30-min period. Following this, all specimens were extracted from the patch. If required, specimens were carefully separated from dry ice by hand, using a small metal ice pick and cool dechlorinated tap water (~6 °C). Control patches were raked for up to three 30-s periods (i.e. for the four rake treatment groups) and allowed to air dry for a 2.5-min period following each raking event. In addition, control patches were also allowed to air dry for a 30-min period following the last raking, or non-raking, event. Control patches were not exposed to any thermal treatments, hot or cold. As before, all specimens were returned to the CT room and left to recover for 24 h, after which mortality was assessed.

### Experiment 3: Effect of Raking with Cold Thermal Shock Treatments, then a Final Hot Shock Application

To investigate the combined impact of cold followed by hot thermal shock treatments on sand encapsulated *C. fluminea*, specimens were exposed to combined applications of non-disrupted and disrupted substrate, dry ice and various hot thermal shock treatments in a fully factorial experiment. As above, the integrity of the substrate was disrupted through raking, while cold thermal shock was achieved with the application of 9 mm dry ice pellets. Hot thermal shock treatments consisted of steam spray or high-intensity open-flame burns. Once again, groups of 30 *C. fluminea* specimens (SH min.–max.: 20–22 mm) were mixed into damp sand patches. Raking periods lasted for 30 s, and were followed by the application of 800 g of dry ice. Dry ice was then evenly spread over the entire patch, with exposure lasting for a 30-min period. Following this, each patch received either no further treatment or a hot thermal shock lasting 2.5 min. All combinations of experimental applications were examined for single, double or triple treatments (*n* = 3 per experimental group). Once the final hot thermal shock was complete, specimens were allowed to cool for a 15-min period before being extracted from the patch. Control patches were raked for up to three 30-s periods and allowed to air dry for a 2.5-min period following each raking event. Control patches were also allowed to air dry for a 15-min period following the last raking event. Control patches were not exposed to any thermal treatments, hot or cold. As above, all specimens were returned to the CT room and left to recover for 24 h after which mortality was assessed. Low-intensity burns were omitted due to a lack of appropriately standardised *C. fluminea* specimens, as well as via an indication that frozen substrate can effectively insulate clams at the low-intensity burns, gleaned from a pre-experimental scoping exercise.

### Data Analyses

Bivalve mortality rates in each experiment were analysed separately according to thermal shock and rake treatments and their interaction, using binomial generalised linear models. Bias reductions were employed for Experiment 3, owing to complete separation of residuals (Firth 1993; Kosmidis 2014). Analysis of deviance with type III sums of squared was used to calculate effect sizes and *P* values (Fox and Weisberg 2011). Tukey tests via estimated marginal means were used to undertake post-hoc pairwise comparisons (Lenth 2018). All analyses were undertaken using the R statistical software environment (R Core Development Team 2018).

## Results

### Experiment 1: Effect of Raking with Hot Thermal Shock Treatments

**Fig. 1:**
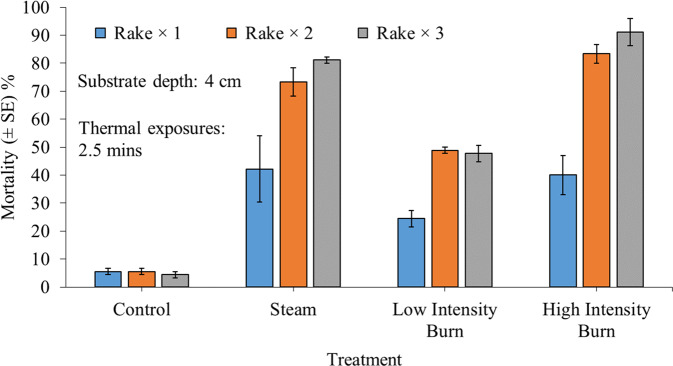
Mean mortality (±SE) of 30 adult *Corbicula fluminea* specimens (480 ind. m^−2^) 24 h following exposure to the application of combined 30-s rake and 2.5-min thermal shock treatments, while residing within a 4 cm deep patch of damp sand substrate. Thermal treatments consisted of steam spray (≥100 °C), low-intensity or high-intensity open-flame exposure (~1000 °C). Although raked for up to three times, control patches were not exposed to thermal treatment. All treatments were performed as a single, double (×2) or triple (×3) applications (*n* = 3 per experimental group)

Whilst control mortality never exceeded 7% across all rake treatments, up to 100% *C. fluminea* mortality was observed following triple rake and thermal shock applications via high-intensity open-flame burn treatments (Fig. 1). Thermal shock and rake treatments interacted significantly (χ^2^ = 19.13, df = 6, *P* = 0.004). Whilst a significant increase in mortality was always induced via hot thermal shock compared to controls (all *P* < 0.01), there was no significant difference among thermal shock treatments following single rake applications (all *P* > 0.05). Contrastingly, after double and triple rake applications, differences emerged among heat treatments. Mortality rates were significantly higher following steam or high-intensity open-flame treatments relative to low-intensity burning after multiple raking treatments (all *P* < 0.01). Although high-intensity open-flame treatments tended to be most effective overall, both steam and high-intensity open-flame always caused similar levels of *C. fluminea* mortality rates (all *P* > 0.05).

### Experiment 2: Effect of Raking with Hot Thermal Shock Treatments, then a Final Cold Shock Application

**Fig. 2:**
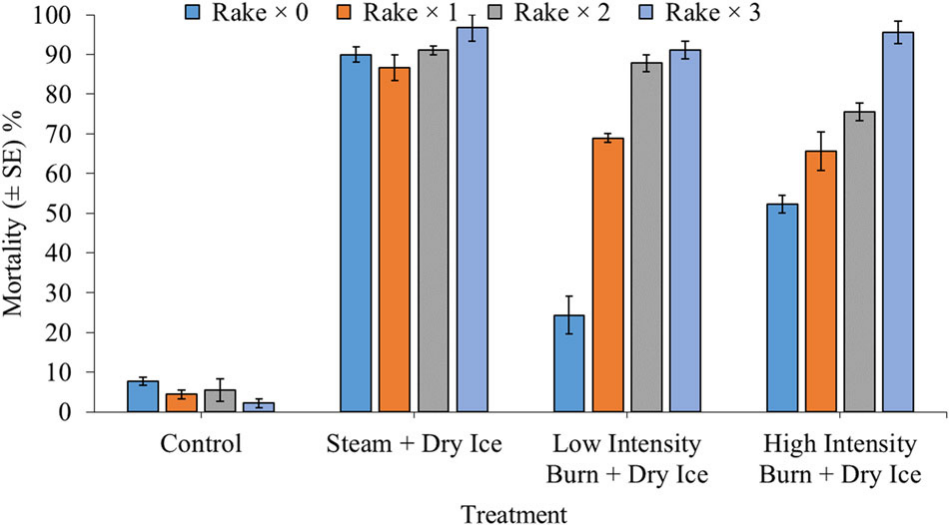
Mean mortality (±SE) of 30 adult *Corbicula fluminea* specimens (480 ind. m^−2^) 24 h following exposure to the application of combined 30-s rake and 2.5-min thermal shock treatments, while residing within a 4 cm deep patch of damp sand substrate. Thermal treatments consisted of steam spray (≥100 °C), low-intensity or high-intensity open-flame exposure (~1000 °C). Patches were evenly covered with 800 g of dry ice for a 30-min period immediately after the final hot thermal shock application. Control patches were not exposed to thermal treatment, hot or cold, but were raked for up to three times. Treatments were performed either in the absence of raking, or with single, double (×2) or triple (×3) applications (*n* = 3 per experimental group)

Whilst control mortality never exceeded 10%, triple steam and open-flame thermal shock treatments, followed by a final dry ice exposure, caused up to 100% mortality of sand dwelling *C. fluminea* (90–100%: Fig. 2). Within combined hot and cold thermal shock application, steam exposures tended to be the most efficacious thermal treatment overall. As before, there was a significant interaction between thermal shock and rake treatments (χ^2^ = 62.94, df = 9, *P* < 0.001). Control mortality was always significantly lower than thermal shock treated groups, irrespective of raking exposure (all *P* < 0.05). In the absence of raking, combined steam and dry ice treatments were significantly more effective in inducing mortality than low- or high-intensity open-flame equivalents (both *P* < 0.001). In turn, high-intensity burning followed by dry ice was more efficacious than low-intensity open-flame equivalents (*P* = 0.001). However, following single, double or triple rake treatments, differences among low- and high-intensity open-flame treatments, followed by dry ice, were not statistically clear (all *P* > 0.05). Hot thermal shock treatments with steam were significantly more effective than both the open-flame treatments for single rake exposures (both *P* < 0.05). However, although steam treatments were more effective than high-intensity burns (*P* = 0.03), they were not more effective than low-intensity burns (*P* = 0.89) under double rake exposures. Conversely, there were no significant differences among thermal shock treatments following triple raking (all *P* > 0.05). Therefore, differences among combined hot and cold thermal shocks were driven by rake treatments, with raking especially enhancing the efficacy of open-flame treatment groups.

### Experiment 3: Effect of Raking with Cold Thermal Shock Treatments, then a Final Hot Shock Application

**Fig. 3:**
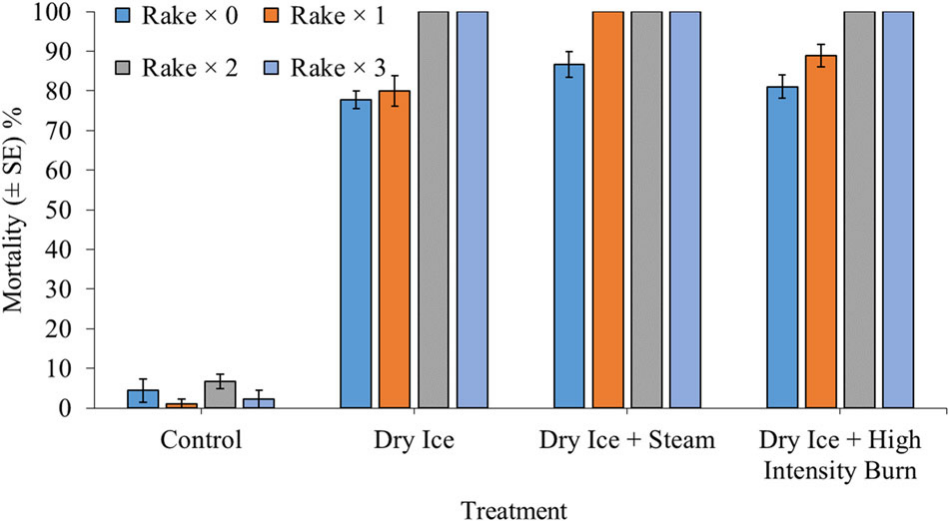
Mean mortality (±SE) of 30 adult *Corbicula fluminea* specimens (480 ind. m^−2^) 24 h following exposure to the application of combined 30-s rake and 2.5-min thermal shock treatments, while residing within a 4 cm deep patch of damp sand substrate. Patches were evenly covered with 800 g of dry ice for a 30-min period prior to the application of each hot thermal shock treatment, which consisted of steam spray (≥100 °C), low-intensity or high-intensity open-flame exposure (~1000 °C). Although raked for up to three times, control patches were not exposed to thermal treatment, hot or cold. Treatments were performed either in the absence of raking, or with single, double (×2) or triple (×3) applications (*n* = 3 per experimental group)

Although exposed to three rake cycles, control specimen mortality never exceeded 10%. However, cold thermal shock via dry ice consistently caused 100% mortality of *C. fluminea* following double and triple applications, irrespective of additional hot thermal shock treatments (Fig. 3). In addition, cold thermal shock treatments followed by a steam application consistently killed all sand dwelling C*. fluminea*, even following just single rake applications. *Thermal shock* and rake effects, again, interacted significantly in causing *C. fluminea* mortality (χ^2^ = 32.85, df = 9, *P* < 0.001). Cold thermal shock with or without hot thermal shock always caused significant mortality relative to controls, irrespective of rake treatment (all *P* < 0.001). Nevertheless, thermal shock treatments were always statistically similar following non-raked, double rake and triple rake applications (all *P* > 0.05). Contrastingly, following single rake treatments, the addition of steam significantly increased *C. fluminea* mortality compared to dry ice alone (*P* = 0.04). Overall, although dry ice was highly efficacious in causing mortality of *C. fluminea* alone, hot thermal shock applications bolstered cold thermal shock impacts under instances with reduced substrate disturbance.

## Discussion

Previous studies have shown that thermal shock treatments caused by dry ice (Coughlan et al. 2018b), steam spray (Coughlan et al. 2019a; Joyce et al. 2019) and open-flame burn applications (Coughlan et al. 2019b; 2020) can be used to rapidly kill substrate-residing *C. fluminea* under laboratory conditions. The present study has confirmed and significantly progressed these observations through a comparative assessment, for applications of substrate disruption (i.e. raking) followed by various combined hot and cold thermal shock treatments. Initially, Experiment 1 highlighted that high-intensity burns are more efficacious than low-intensity burning, especially following multiple applications, in advance of the combined application of hot and cold thermal treatments in Experiments 2 and 3. Interestingly, steam spray treatments were also highly effective. Further, Experiment 2 revealed that the application of hot and cold (i.e. dry ice) thermal shock treatments can substantially increase *C. fluminea* mortality. Once again, steam and high-intensity open-flame burns were generally observed to cause greater mortality than low-intensity burns. In particular, the efficacy of raking and multiple thermal shock applications was clearly evidenced, and particularly for open-flame treatments. Building on this, Experiment 3 demonstrated that the application of dry ice, especially when combined with substrate raking over multiple treatments, can effectively kill sand dwelling *C. fluminea*. Comparatively, Experiments 2 and 3 show that when cold thermal shock treatments are followed by the application of a hot thermal shock, a greater proportion of *C. fluminea* can be killed with a single exposure, with 100% mortality being achievable with two or fewer applications of combined rake and thermal shock treatments.

As previously described by Coughlan et al. (2020), groups of both surface-dwelling and buried *C. fluminea* can be completely killed, following 1- or 5-min steam exposures, respectively, (as per Figure 2 in Coughlan et al. op. cit.). Similarly, open-flame heat torch treatments can be used for effective and substantial control of *C. fluminea* populations, especially when combined with the prior disruption of substrate integrity to bring more specimens towards the surface (Figure 5 in Coughlan et al. 2019b; Figure 4 in Coughlan et al. 2020). Although not statistically clear, Coughlan et al. (2020) observed that high-intensity open-flame burn treatments tended to kill marginally more *C. fluminea* than steam applications (Figure 4 in Coughlan et al. op. cit.), and this is thought to be most likely due to the more intense heat generated by open-flame relative to steam, i.e. ~1000 and ~100 °C, respectively. In the present study, which sought to ascertain the effects of combined hot and cold thermal shock treatments over a wider temperature gradient, steam appears to have an equal and sometimes greater efficacy than high-intensity open-flame burns when combined with an application of dry ice. A similar trend was observed in Fig. 4 in Coughlan et al. (2020). As steam tends to condense onto substrate as hot water, this heated water may penetrate through the patch to warm and further saturate encapsulated *C. fluminea*. Subsequent addition of dry ice will then rapidly cool and freeze the substrate causing thermal shock (see Coughlan et al. 2018b), through a sudden change over a wide temperature range. Indeed, in Experiment 2, it was difficult to extract *C. fluminea* from patches following a final treatment of dry ice as the substrate froze around the specimens. Similarly, the application of steam condensing into hot water, following an initial dry ice treatment, likewise causes a sudden temperature change within experimental patches. In contrast, substrate appears to insulate buried *C. fluminea* from open-flame treatments, which tends to dry and solidify the sand substrate (see Coughlan et al. 2020).

Overall, we suggest that applications of raking combined with both cold and hot thermal shock treatments could be used as a rapid-response tool to control emerging and established populations of *C. fluminea* found residing at dewatered locations, such as exposed river, lake and canal beds. In particular, it appears that cold thermal shock treatments followed by hot thermal applications of steam or high-intensity open-flame burn would likely be the most effective approach. In the absence of substrate disturbance measures, steam followed by dry ice appears to be the most efficacious means of control. However, further validation and clarification of these techniques is required. For example, the impact of deeper substrate depths will likely reduce the efficacy of thermal shock treatments (Coughlan et al. 2020), and this will need to be considered in greater detail. Further, although completely water-saturated sand substrate can reduce the efficacy of rake and steam treatments (Coughlan et al. 2020), the application of dry ice to cause a freezing effect may further increase mortality rates in such scenarios. However, even though dry ice-induced cold thermal shock treatments have been shown to effectively kill *C. fluminea* at a submerged water depth of up to 10 cm (Coughlan et al. 2018b), this has not yet been examined under field conditions. Hitherto, field-tested underwater mechanical control methods for bivalve populations, such as benthic barriers and dredging, have proven problematic, costly and unreliable (see, e.g. Wittmann et al. 2012a, b; Sheehan et al. 2014). However, as argued by Coughlan et al. (2020), thermal shock treatments could be combined with mechanical dredging methods to improve bivalve population management strategies. In turn, such treatments may enhance waste disposal practices, effectively ensuring any extracted *C. fluminea* are killed prior to final disposal. Further, physiological responses to elevated temperatures can include degeneration of the gill filaments and detrimental damage of internal organs (Gonzalez and Yevich 1976), which can contribute to overall population morbidity and mortality (White et al. 2015). Nevertheless, the tested approaches cannot be used to eradicate clams residing in locations that remain under water. Accordingly, there remains a pressing need to develop in-water control techniques for *C. fluminea*.

Whilst the results presented herein are promising, additional research is therefore needed to confirm the effectiveness of multiple thermal shock treatments under natural field conditions. Although it is likely that rapid thermal shock applications will adversely impact non-target species over relatively short timescales, these effects may be outweighed by long-term conservation benefits associated with invader eradications (Woodford et al. 2013), and particularly of those invasions which homogenise communities. Lotic systems in particular benefit from high levels of biological connectivity and are often recolonised rapidly through, e.g. drift from uninvaded upstream refuges (Wittmann et al. 2012a, b; Coughlan et al. 2017a; Bellingan et al. 2019). These factors may thus mitigate any long-term ecological impacts of these methods. Nevertheless, thorough assessment of direct and indirect thermal shock treatment effects on biodiversity, such as mortality of native species within *C. fluminea* beds and inconspicuous decomposition effects, is required.

Although the application of combined thermal shock treatments could be expensive and laborious, given the current lack of effective and environmentally friendly invader eradication and control protocols, the excellent potential shown by these innovative treatments requires further investigation under different environmental contexts, such as for specimens residing at substrate depths in excess of 4 cm. Particularly as *C. fluminea* residing deeper within the substrate may escape treatment and subsequently facilitate population recovery to the pre-treatment level. Further, a comparative assessment of financial costs of the proposed treatments, current control practices and the cost of inaction should be undertaken. Nonetheless, studies have shown that preventative measures are much more cost-effective compared to longer-term control (Leung et al. 2002). Whilst thermal shock treatments will incur an expense, this may be relatively more affordable than other labour-intensive management strategies, such as harvesting, dredging and benthic barriers, which have largely been found to ineffective for control of *C. fluminea* populations (Wittmann et al. 2012a, b; Sheehan et al. 2014). In addition, thermal treatments may be especially useful if they provide for rapid-reaction and long-term population control of problematic bivalve infestations, such as those residing in raw water intake/extraction sites associated with power stations, potable water treatment plants and other raw water using industries. Indeed, the financial cost of treatment applications may be more readily justifiable at commercial sites, as opposed to natural settings. Nevertheless, it appears that 100% mortality can be readily achieved with an application of cold thermal shock (dry ice) followed two or fewer applications of combined rake and 2.5-min hot thermal shock treatments. This considerably advances the previous works by Coughlan et al. (2019b; 2020), which found that three bouts of combined rake and 5-min hot thermal shock treatments were required to achieve 100% mortality. Overall, although in situ confirmation is required, it is argued that the application of hot and cold thermal shock treatments could represent a method for control and possible eradication of *C. fluminea*.
